# Nanocarriers for DNA Vaccines: Co-Delivery of TLR-9 and NLR-2 Ligands Leads to Synergistic Enhancement of Proinflammatory Cytokine Release

**DOI:** 10.3390/nano5042317

**Published:** 2015-12-17

**Authors:** Johanna Poecheim, Simon Heuking, Livia Brunner, Christophe Barnier-Quer, Nicolas Collin, Gerrit Borchard

**Affiliations:** 1 School of Pharmaceutical Sciences, University of Geneva, University of Lausanne, Quai Ernest Ansermet 30, 1211 Geneva, Switzerland; E-Mail: johanna.poecheim@unige.ch; 2 Vaccine Formulation Laboratory, Department of Biochemistry, University of Lausanne, Chemin des Boveresses 155, 1066 Epalinges, Switzerland; E-Mails: simon.heuking@gmx.net (S.H.); livia.brunner@unil.ch (L.B.); christophe.barnier-quer@unil.ch (C.B.-O.); nicolas.collin@unil.ch (N.C.)

**Keywords:** adjuvants, toll-like receptor, NOD-like receptor, cationic nanoparticles, DNA vaccine, muramyl dipeptide

## Abstract

Adjuvants enhance immunogenicity of vaccines through either targeted antigen delivery or stimulation of immune receptors. Three cationic nanoparticle formulations were evaluated for their potential as carriers for a DNA vaccine, and muramyl dipeptide (MDP) as immunostimulatory agent, to induce and increase immunogenicity of *Mycobacterium tuberculosis* antigen encoding plasmid DNA (pDNA). The formulations included (1) trimethyl chitosan (TMC) nanoparticles, (2) a squalene-in-water nanoemulsion, and (3) a mineral oil-in-water nanoemulsion. The adjuvant effect of the pDNA-nanocomplexes was evaluated by serum antibody analysis in immunized mice. All three carriers display a strong adjuvant effect, however, only TMC nanoparticles were capable to bias immune responses towards Th1. pDNA naturally contains immunostimulatory unmethylated CpG motifs that are recognized by Toll-like receptor 9 (TLR-9). In mechanistic *in vitro* studies, activation of TLR-9 and the ability to enhance immunogenicity by simultaneously targeting TLR-9 and NOD-like receptor 2 (NLR-2) was determined by proinflammatory cytokine release in RAW264.7 macrophages. pDNA in combination with MDP was shown to significantly increase proinflammatory cytokine release in a synergistic manner, dependent on NLR-2 activation. In summary, novel pDNA-Ag85A loaded nanoparticle formulations, which induce antigen specific immune responses in mice were developed, taking advantage of the synergistic combinations of TLR and NLR agonists to increase the adjuvanticity of the carriers used.

## 1. Introduction

Vaccination by direct injection of antigen encoding plasmid DNA (pDNA) has been evaluated for decades. Several preclinical studies have revealed that DNA vaccines can trigger not only humoral but also cell-mediated immunity in animals [1]. DNA vaccines usually consist of a bacterial plasmid vector genetically modified to express selected antigens of the pathogen in the absence of the other bacterial components present in traditional live or attenuated vaccines. However, although proven to be usually safe and well tolerated in clinical trials, first-generation DNA vaccines failed to demonstrate sufficient vaccine-specific immunogenicity in humans [2,3,4]. One hypothesis for the low immunogenicity of DNA vaccines is suboptimal delivery of the plasmids to antigen presenting cells (APCs). Current research focuses on developing novel strategies to improve immunogenicity by formulating pDNA with delivery systems and novel specific adjuvants [5,6]. Among these systems, particle-based adjuvants of high loading capacity act as delivery vehicles for pDNA to enhance plasmid stability and uptake into cells [7,8]. Moreover, versatility of particulate carriers in terms of size, surface charge, and material used, enables tailoring toward the desired outcome of immune responses [9,10,11]. Another potential advantage of nano- and microparticles is the induction of local chemotaxis to the immunization site due to the promotion of a depot effect [12]. Adsorbing pDNA at the outer surface of diverse cationic nanoparticles may lead to the avoidance of steric shielding effects by pDNA encapsulation, while conserving the adjuvant effects of nanoparticles.

Three cationic nanocomplexes offering different structural characteristics were chosen for this study: *N*-trimethyl chitosan (TMC) nanoparticles, a cationic squalene-in-water nanoemulsion (named SWE06), and the commercially available cationic nanoemulsion, Cationorm^®^. These particles were compared for their adjuvant potential to increase immunogenicity of pDNA in mice. Chitosan-based particles have been used previously as DNA delivery systems, with pDNA entrapped inside the nanoparticles, maintaining the cationic surface charge, which may be beneficial for mucosal application of DNA vaccines [13,14]. However, previous studies in our laboratory using such formulations did not demonstrate higher immunogenicity over non-adjuvanted pDNA, when administered intramuscularly in mice. The strong electrostatic charge interaction of condensed pDNA with the cationic polymer may interfere with DNA release once the polyplex is taken up by the target cells.

Squalene based oil-in-water emulsions have already been approved for human use in influenza vaccines and are well characterized [15,16]. The cationic phospholipid 1,2-dioleoyl-3-trimethylammonium-propane (DOTAP) was added to the hydrophobic phase of the squalene-in-water nanoemulsion (SWE06) to obtain positively charged oil droplets. Cationorm^®^ obtained a marketing authorization in Europe for the treatment of dry eye syndrome and was chosen as a third type of nanoformulation in our studies. It is known to be safe in ophthalmic applications and was therefore considered as a representative example for inert mineral oil-in-water nanoemulsions, with cetalkonium chloride accounting for the positive charge [17].

DNA vaccines promote exogenous major histocompatibility complex (MHC) class II-restricted, as well as endogenous MHC class I-restricted antigen presentation. The latter process mimics antigen processing induced by intracellular pathogens such as *Mycobacterium tuberculosis* (*Mtb*) [18], against which cellular immunity, including proinflammatory cytokines and Th1 cells are believed to play a pivotal role. Among Th1 cytokines, interferon gamma (IFN-γ) and tumor necrosis factor alpha (TNF-α) have been identified as the most important agents in mycobacterial control, acting synergistically in the activation of macrophages [19,20]. The innate immune system has evolved to recognize conserved pathogen-associated molecular patterns (PAMPs) by pattern recognition receptors (PRRs). These are mainly represented by Toll-like receptors (TRLs) and NOD-like receptors (NLRs), which contribute to the host’s ability to eliminate the pathogen. PRR stimulation activates the production of proinflammatory cytokines that possess immunoregulatory functions by bridging innate resistance and antigen-specific adaptive immune responses [21]. Recognition of bacteria, as well as vaccination with live attenuated vaccines, induces activation of multiple PRRs, triggering different signaling pathways, which has been shown to be more effective in establishing immune responses than activation of a single pathway alone [22]. Unmethylated CpG sequences present on bacterial pDNA are recognized by endosomal membrane-bound TLR-9, whereas muramyl dipeptide (MDP), a bacterial cell wall component, stimulates cytosolic NLR-2. TLR-9 activates the MyD88-dependent pathway, while NLR-2 activation leads to recruitment of receptor-interacting protein 2 (RIP-2) kinase [23,24]. There is documentation on cross-talk of TLR and NLR signaling through RIP-2, demonstrating extensive activation of immune cells in a synergistic manner by simultaneous co-activation of these two pathways [25,26,27,28].

In an *in vivo* experiment in mice we compared TMC nanoparticles, SWE06, and Cationorm^®^ as pDNA delivery systems to increase Th1 related immune responses against Ag85A. Following these investigations, we then exploited the potential of concurrent activation of two non-redundant PRR pathways *in vitro* with the aim of further optimizing immunogenicity of pDNA. Our results show that cationic TMC nanoparticles are promising carriers for pDNA and co-delivery with MDP can be used to further increase immunogenicity of this DNA vaccine formulation.

## 2. Results and Discussion

### 2.1. Nanoparticle Characterization

The formulations were characterized for their size by differential laser light scattering (DLS) expressed as *Z*-average (nm), polydispersity index (PDI), and their charge as zeta potential (mV), as shown in Table 1. The mean hydrodynamic diameter of the unloaded polymer complexes and nanodroplets was between 133 and 216 nm (PDI ≤ 0.2) and their surface charge was positive, ranging between +18 and +31 mV. After adsorption of pDNA, the size increased for TMC nanoparticles (*p* < 0.001) and both nanoemulsions (*p* < 0.0001), while the zeta potential decreased drastically to 7 mV (TMC nanoparticles), −14 mV (SWE06) and −39 mV (Cationorm^®^). The addition of MDP did not have any influence on size and zeta potential of TMC nanoparticles and SWE06. However, size increase and higher PDI values of pDNA loaded Cationorm^®^ with MDP indicated aggregation tendencies of this formulation. PDIs between 0.1 and 0.5 were observed for all particles, corresponding to systems of mid-range polydispersity [29]. Only small amounts of pDNA were found in the supernatant, having measured pDNA adsorption of 99.8% to TMC nanoparticles, 95% to SWE06, and 93% to Cationorm^®^ of initially added 50 μg/mL pDNA to the cationic nanocomplexes.

**Table 1:** Physicochemical properties of trimethyl chitosan (TMC) nanoparticles, a cationic squalene-in-water nanoemulsion (SWE06) and Cationorm^®^, either unloaded or loaded with pDNA, muramyl dipeptide (MDP), or both. To determine size in nm, polydispersity index (PDI), and zeta potential (ζ) in mV, samples were prepared in water and diluted with 1 mM NaCl prior to measurements.

Loaded with	TMC Nanoparticles			SWE06			Cationorm^®^		
	Size (nm)	PDI	ζ (mV)	Size (nm)	PDI	ζ (mV)	Size (nm)	PDI	ζ (mV)
(blank)	216 ± 2	0.1	31 ± 2	133 ± 2	0.1	27 ± 1	157 ± 3	0.2	18 ± 1
pDNA	252 ± 5	0.2	7 ± 5	188 ± 3	0.1	−14 ± 1	205 ± 2	0.3	−39 ± 0.5
MDP	215 ± 0.5	0.2	32 ± 4	134 ± 2	0.2	26 ± 1	156 ± 2	0.2	14 ± 0.2
pDNA and MDP	259 ± 3	0.2	4 ± 5	189 ± 3	0.2	−16 ± 1	267 ± 12	0.5	−41 ± 1

**Figure 1:**
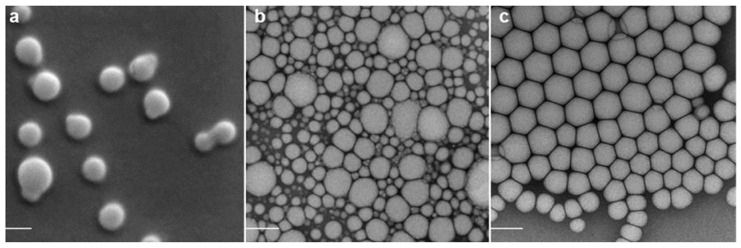

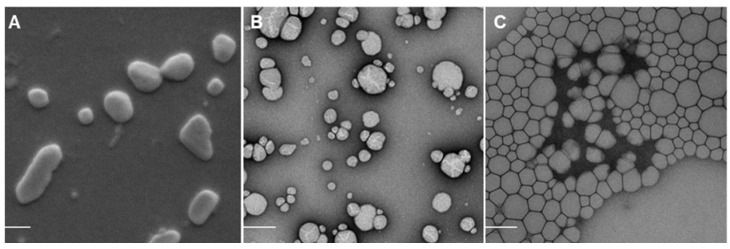
Scanning electron microscopy (SEM) images of plain TMC nanoparticles alone (**a**) and loaded with pDNA (**A**), transmission electron microscopy (TEM) images of SWE06 alone (**b**) and loaded with pDNA (**B**), and TEM images of Cationorm^®^ alone (**c**) and loaded with pDNA (**C**). The scale bars represent a size of 200 nm.

Particle size calculations from electron microscopy imaging confirmed the measurements of hydrodynamic diameters of empty nanocarriers by DLS (Figure 1). Mean diameters determined by electron microscopy were slightly smaller than determined by DLS (192 ± 28 nm for TMC nanoparticles, 114 ± 37 nm for SWE06 and 126 ± 21 nm for Cationorm^®^). This might be due to shrinking during preparation, which can affect the measurements of particle diameters [30]. TMC nanoparticles were of globular shape (Figure 1a), whereas the emulsions droplets were of subangular shape (Figure 1b,c). Upon pDNA adsorption TMC nanoparticles appeared to deform by loosening the electrostatically formed structure (Figure 1A). pDNA at the external site of nanoemulsion droplets make them distract, presumably due to charge repulsion, resulting in appearance of single droplets and small groups of droplets (Figure 1B). Repulsion was also observed within pDNA loaded Cationorm^®^, however to a lesser extent (Figure 1C).

### 2.2. Adjuvant Effect of pDNA-Nanoformulations in Vivo

In this first experiment, performed in mice, we evaluated TMC nanoparticles, SWE06 and Cationorm^®^ applied with *Mtb* antigen Ag85A encoding pDNA for their potential to increase *Mtb* antigen-specific Th1 related immune responses of a tuberculosis DNA vaccine candidate. Ag85A possess enzymatic mycolyltransferase activity involved in cell wall synthesis and belongs to the key immunodominant antigens of Mtb. We decided to apply the same formulation preparations as described above but with a higher quantity of pDNA applied (50 μg per dose) to ensure a detectable magnitude of antigen-specific antibodies. The influence of the nature of the nanocomplexes on the outcome of elicited immune responses in mice, dependent on the nature of the delivery systems was evaluated. The loading efficiency of pDNA to the nanoparticles within these formulations was 43% to 44%, while surplus pDNA remained in suspension.

**Figure 2:**
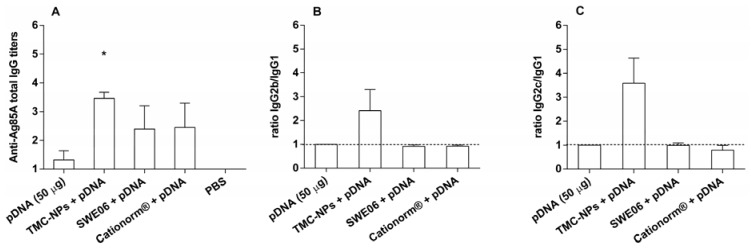
Immune responses in mice to pDNA (50 μg per dose) with/without TMC nanoparticles one week after the second booster injection (i.m.). Ag85A-specific serum immunoglobulin G (IgG) titers were analyzed by endpoint enzyme-linked immunosorbent assay (ELISA). (**A**) Bars represent mean *n* = 4 ± SEM, * *p* < 0.05, compared to pDNA alone. (**B**,**C**) Corresponding average Log IgG2b/Log IgG1 and Log IgG2c/Log IgG1 ratios are indicative for the quality of the immune response, where values higher than 1 (dotted line) characterize Th1 biased immune responses.

Antigen specific total IgG responses to pDNA in the adjuvanted groups were higher than those observed for naked pDNA. In TMC nanoparticle vaccinated mice significantly increased titers of total IgG were observed in comparison with pDNA alone, as shown in Figure 2A (*p* < 0.05). Oil-in-water emulsions based on squalene or mineral oils reportedly induce Th2 responses in protein vaccines [31,32]. Formulated with DNA both nanoemulsions tested promoted increase in Ag85A specific antibodies to pDNA without altering the balanced Th1/Th2 responses observed with naked pDNA (Figure 2).

TMC nanoparticle adjuvanted pDNA delivery induced a clear bias of Th activation towards type 1, which was indicated by detection of ratios of serum IgG2b/IgG1 (Figure 2B) and IgG2c/IgG1 (Figure 2C) titers above unity. According to the literature, the adjuvant effect of TMC and chitosan on Th1/Th2 balance seems to be highly dependent on the antigen applied, route of administration, or formulation as particles [33]. Chitosan itself reveals Th2 adjuvant effects [34,35], which are shifted towards Th1 when formulated as particles [36,37]. Moreover, although we did not investigate the physical interaction between CpG containing pDNA, a TLR-9 ligand, and TMC, the physicochemical properties of chitin derivatives, known to activate PRRs such as TRL-2, Dectin-1, and the mannose receptor could mediate such interactions [38]. Presumably increase in Th1 type responses is dependent on the triggering of distinct intracellular signaling MyD88 dependent and independent pathways, which then merge to activate NF-κB, as has been demonstrated following stimulation of mannose receptors and TLR-2 in parallel to TLR-9 stimulation [39]. Whether this Th1 polarization results from the physical association of pDNA with TMC or the effect of enhanced uptake of pDNA through particle mediated delivery, remains to be determined. Our experiments suggest that formulation of pDNA with adjuvants may be necessary to impart Th1 adjuvanticity.

### 2.3. Cell Viability

**Figure 3:**
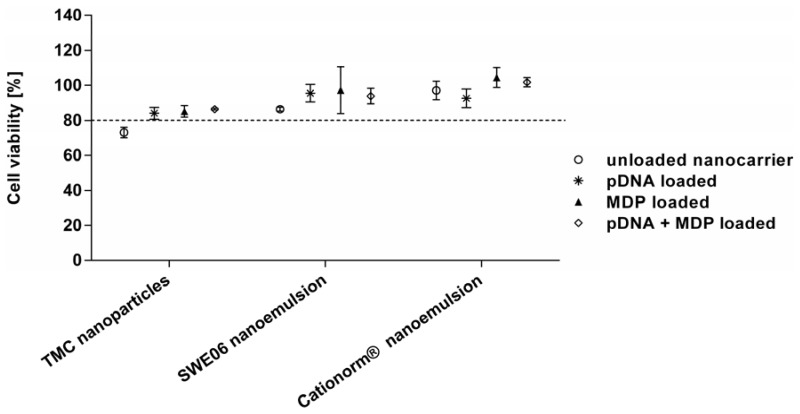
Cell viability of RAW264.7 macrophages, detected with XTT reagent, after 24 h of incubation with the following formulations: unloaded TMC nanoparticles, SWE06 and Cationorm^®^ (∘) and each nanoformulation loaded either with pDNA (∗), MDP (▲), or pDNA + MDP (⋄). Percentages above 80% (dotted line) were considered as minimum levels of acceptable viability.

Cell viability after exposure to the different particle formulations for 24 h in culture were confirmed with XTT assay including Trypan blue dye exclusion, as shown in Figure 3. It has been proposed that cationic nanoparticles interact with the cell membrane and may cause damage by membrane disruption, leading to cell death [40]. Therefore investigation of the potential toxicity of the cationic nanoformulations is important in order to exclude biological responses due to cell death and to assess safety concerns for future *in vivo* applications. When uncomplexed TMC polymer was applied to the cells, reduced cell viability was observed (data not shown). Chondroitin sulfate partially balanced the cationic charge of TMC and, as a consequence, cell viability to the resulting nanoparticles increased. This is in accordance with other studies that showed less toxicity upon charge neutralization with anionic agents or pDNA [41,42].

No significant toxicity was detected for any of the nanoformulations, either unloaded, pDNA and/or MDP loaded, compared to the untreated control cells (viability >80% in all cases). For TMC nanoparticles and SWE06 emulsions cell viability was slightly increased with pDNA loading compared to the unloaded formulations, which may be due to neutralization of cationic surface charges known to be linked to better biocompatibility [43].

### 2.4. In Vitro Activation of TLR-9 and NLR-2

**Figure 4:**
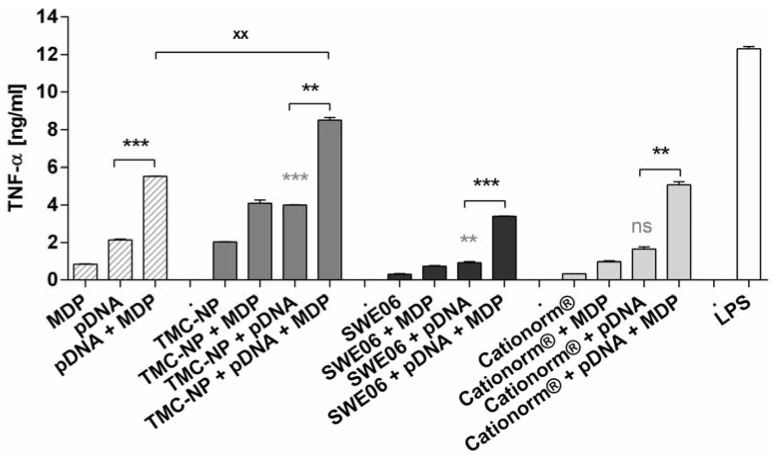
Tumor necrosis factor-alpha (TNF-α) release from RAW264.7 murine macrophages on exposure to different stimulating agents: pDNA and MDP applied either alone or in combination in solution, or with TMC nanoparticles (TMC-NP), SWE06, and Cationorm^®^, respectively. Bars represent mean values (*n* = 3) ± SEM. pDNA loaded nanoformulations were compared with either pDNA alone (*) or with [pDNA + MDP] loaded nanoformulations (*), and [pDNA + MDP] in solution with [nanocarrier + pDNA + MDP] (x). Significant differences were indicated with ** (*p* < 0.01), *** (*p* < 0.001), and ns (not significant).

In subsequent mechanistic experiments on RAW264.7 murine macrophages the inherent adjuvant effect of unmethylated CpG containing pDNA in combination with MDP as an additional immunostimulator, delivered with all three cationic nanocomplexes, was investigated to potentially further optimize cell-mediated immunity by simultaneous innate immune activation. pDNA adsorbed to nanoparticles and nanoemulsions were formulated with MDP and studied for their ability to release proinflammatory cytokine TNF-α. Synergistic enhancement of immune response by two PRR ligands was investigated using CpG islet containing pDNA for TLR-9 stimulation, and MDP as a NOD2 ligand. Emerging evidence suggests cooperative effects of PRRs [44]. Consequently, we also investigated the outcome of combined stimulation of the TLR and NLR systems. Neither pDNA nor MDP alone could substantially activate macrophages as shown in Figure 4. TMC nanoparticles loaded with pDNA were shown to significantly increase cytokine release, compared to pDNA alone (*p* < 0.001). Carrier function for Cationorm^®^ appeared to be less important and SWE06 even decreased TNF-α release induced by pDNA alone (*p* < 0.01). When we combined both ligands in one formulation, the NOD2 ligand MDP augmented pDNA-induced activation of murine macrophages *in vitro* by up to 4-fold, with a significant increase of TNF-α release compared to naked pDNA (*p* < 0.001) or pDNA adsorbed to nanocarriers (TMC nanoparticle *p* < 0.01, SWE06 *p* < 0.001, Cationorm^®^ *p* < 0.01). This indicates that MDP has an influence on enhancing immune responses in a synergistic manner with the TLR ligand, as cytokine production by the combination of immune receptor ligands was higher than production of each of the single stimulations and as the sum of each effect individually. The adjuvant function of TMC nanoparticles seemed to be most important for the co-delivery of pDNA and MDP in one formulation. TMC nanoparticles were the only carriers for pDNA and MDP that were shown to be significantly superior in stimulating cytokine release when compared to the ligands combined in solution (*p* < 0.01).

**Figure 5:**
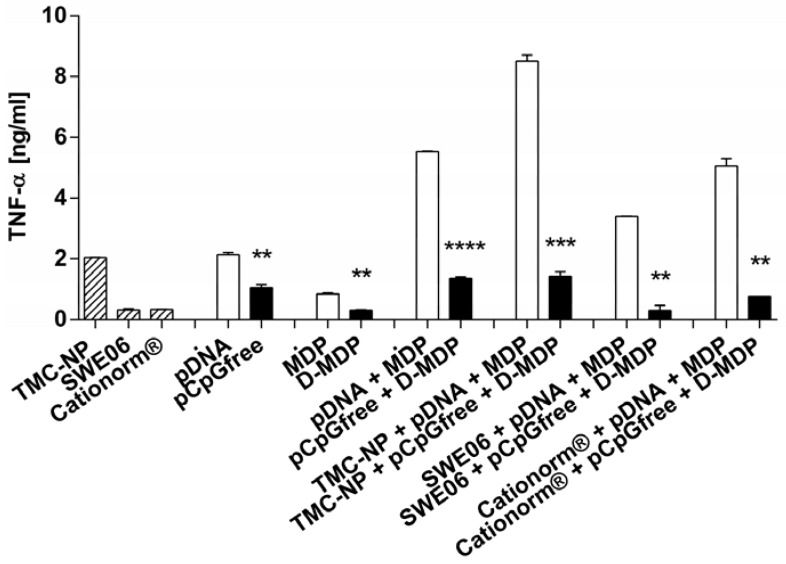
TNF-α release from RAW264.7 murine macrophages treated with pDNA and/or MDP and their inactive controls pCpGfree and/or D-MDP, respectively. The ligands were applied either in solution, as single components, or combined with TMC nanoparticles (TMC-NP), SWE06, or Cationorm^®^, respectively. Significantly reduced activity by control ligands compared to their active ligands are indicated with ** (*p* < 0.01), *** (*p* < 0.001), and **** (*p* < 0.0001).

TLR-9 specifically recognizes unmethylated or hypomethylated CpG islets, prevalent in bacterial and many viral DNAs [45]. To confirm that TNF-α response is dependent on TLR-9 activation, macrophages were stimulated with pDNA either containing or lacking CpG motifs. To further assure that the cytokine response is also dependent on NLR-2 activation the inactive D-isoform of MDP was applied in the same experiment. The dependency on PRR activation to induce a distinct TNF-α response towards TLR-9 and NLR-2 ligands implemented in the experiments is shown in Figure 5. Nanoparticles loaded with inactive control ligands were found to not induce cytokine release, compared to the unloaded nanocarriers. Significant decrease of cytokine release was observed for inactive control ligands transported by TMC nanoparticles, SWE06 and Cationorm^®^, compared to their active forms. Cells were minimally stimulated with TMC and CpG-free pDNA containing inactive control samples. This could be due to inherent adjuvant effects of TMC that possibly derives from the chitosan’s molecular features to activate PRRs [46]. Furthermore, there may be potential contributions to immune activation by pDNA by other elements than TLR-9 activation via unmethylated CpG, such as cytosolic TANK-binding kinase-1 activation by the double stranded B-form of pDNA [47]. Mineral oil based incomplete Freund’s adjuvant as well as squalene-based formulations have been shown to polarize the immune response toward the production of anti-inflammatory cytokines [48,49]. This may mask the TNF-α increasing effect by the double stranded nature of CpG-free pDNA, as well as of the active ligands, applied with SWE06 and Cationorm^®^, respectively.

### 2.5. NLR-2 Dependent Synergistic Enhancement of TNF-α Release

**Figure 6:**
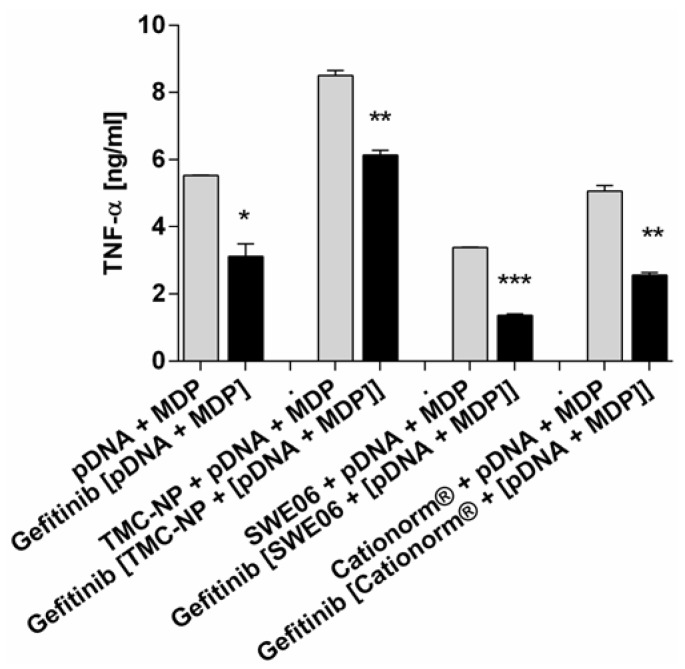
TNF-α release from RAW264.7 murine macrophages treated with or without RIP2 tyrosine kinase blocker gefitinib. Stimulants were pDNA and MDP in combination, applied either in solution or with TMC nanoparticles, SWE06, or Cationorm^®^, respectively. Significantly reduced activity by receptor-interacting serine/threonine-protein kinase 2 (RIP2) blocking compared to unblocked NLR-2 pathway are indicated with * (*p* < 0.05), ** (*p* < 0.01), and *** (*p* < 0.001).

NLR-2 activation was inhibited in order to demonstrate its influence during co-stimulation with TLR-9 ligand on the synergistic enhancement of immune responses. Cells incubated with pDNA and MDP were treated with or without RIP-2 tyrosine kinase inhibitor gefitinib, to study NOD2 synergy with pDNA. The mechanism of the cross-talk between the pathways for PRRs is not yet fully understood, but it has been shown that there are functional links between protein RIP2 and both pathways of TLR and NLR (Chin 2002, Kobayashi 2002). If synergistic enhancement is dependent on interaction between RIP2, activated by NOD2, and MyD88 of the TLR pathway, then inhibition of RIP2 kinase may serve to correct the excessive activation seen with combination of pDNA + MDP [50]. Indeed, we showed that RIP2 blocking led to significant reduction of cytokine release, leading to reduced cytokine release for ligands applied in solution (*p* < 0.05), for TMC nanoparticle-conjugates (*p* < 0.01) and for ligands applied with SWE06 (*p* < 0.001) and Cationorm^®^ (*p* < 0.01) nanoemulsions, respectively, compared to untreated cells (Figure 6). The diminished macrophage activation was shown to be similar to that induced by pDNA alone or pDNA applied with the nanocarriers without MDP.

## 3. Experimental Section

### 3.1. Materials

Chitosan (ChitoClear Cg10, 79% degree of deacetylation, 7–15 mPa·s) was purchased from Primex (Siglufjordur, Iceland). Methyl iodide, chondroitin sulfate and bovine serum albumin (BSA, endotoxin <0.1 ng/mg) were obtained from Sigma-Aldrich (Buchs, Switzerland). DOTAP was purchased from Avanti Polar Lipids (Alabaster, AL, USA) and Cationorm^®^ (Novagali Pharma, Evry, France) was purchased at a local pharmacy. Double stranded 5853 bp pDNA encoding Ag85A was provided by the Institute Pasteur (Brussels, Belgium), propagated in NovaBlue competent cells (Merck, Darmstadt, Germany) that were kindly provided by Scapozza (University of Geneva, Geneva, Switzerland). The plasmid was then purified by using an Endofree Plasmid Giga Kit (Qiagen, France) according to the manufacturer’s protocol and suspended in PBS. Goat anti-mouse total IgG, IgG1, IgG2b and IgG2c conjugated to horseradish peroxidase (HRP) was obtained from Southern Biotech (Birmingham, AL, USA) and 3,3′,5,5′-Tetramethylbenzidine (TMB) substrate from Becton Dickinson (San Diego, CA, USA). All cell culture reagents were provided by Life technologies (Zug, Switzerland) and Sigma-Aldrich (Buchs, Switzerland), and XTT-cell proliferation kit II was bought from Roche (Basel, Switzerland). MDP, as well as LPS-EB, CpG-free pDNA (pCpGfree-giant) and inactive MDP (D-MDP) as controls, and RIP-2 tyrosine kinase inhibitor gefitinib were purchased from Invivogen (San Diego, CA, USA). For proinflammatory cytokine detection in cell supernatants, mouse TNF-α ELISA Ready-SET-Go! Kit was obtained from eBioscience (Paris, France).

### 3.2. Preparation of Nanoparticle Formulations

*TMC nanoparticles:* TMC was synthesized from chitosan by quaternisation of the amino groups based on the method first published by Domard *et al.* [51], and later modified by Heuking *et al.* [52]. In this procedure only one methylation step was employed for the synthesis of TMC. Briefly, 2 g of high molecular weight chitosan of crustacean origin were trimethylated through nucleophilic substitution by addition of 12 mL methyl iodide for 70 min at 60 °C under reflux. TMC nanoparticles were prepared by polyelectrolyte complexation with chondroitin sulfate according to Schatz *et al.* [53]. TMC (5 mg/mL) and chondroitin sulfate (1 mg/mL) were solubilized separately in water at room temperature and filtered through 0.22 μm syringe filters (Millipore AG, Zug, Switzerland). Then, 0.5 mL of each polymer solution was mixed, briefly vortexed at high speed, and the resulting nanoparticle suspension diluted 1:10 with the appropriate media. In case of nanoparticles containing MDP, the dipeptide was mixed with the TMC solution before particle formation. The final MDP concentration per ml of nanoparticle suspension was 30 μg. For pDNA adsorption, 50 μg/mL of the plasmid were incubated for 15 min at 4 °C with the TMC nanoparticle suspensions.

*SWE06 and Cationorm^®^ nanoemulsions*: SWE06, a cationic squalene-in-water nanoemulsion, was manufactured by the Vaccine Formulation Laboratory at the University of Lausanne (Epalinges, Switzerland) and contained 0.1% DOTAP, 3.9% (*w*/*v*) squalene, 0.5% (*w*/*v*) Tween^®^ 80 and 0.5% (*w*/*v*) Span^®^ 85 in saline. Cationorm^®^ was developed by Novagali Pharma (now Santen), an oil-in-water nanoemulsion composed of 0.1% poloxamer 188, 0.3% tyloxapol, 0.002% cetalkonium chloride, 1% mineral oils and 1.6% glycerol in Tris hydrochloride buffer as indicated in the decision report of the French National Authority for Health [54]. Both nanoemulsion formulations were diluted at a ratio of 1:100 with the appropriate media prior to each experiment. pDNA was incubated for 15 min at 4 °C with the diluted nanoemulsions to obtain electrostatic binding of pDNA to the cationic nanodroplets and a final pDNA concentration of 50 μg/mL. For the MDP-containing nanoemulsion formulations, the dipeptide was simply mixed with the diluted emulsion to obtain a final concentration of 30 μg/mL, then vortexed at high speed, and immediately applied in the experiments.

All three nanoparticle suspensions (TMC, SWE06 and Cationorm^®^) showed an endotoxin level <1 EU/mL as tested by Endosafe^®^ Test Record.

### 3.3. Characterization of Nanoparticles

#### 3.3.1. Particle Size, Zeta Potential and Morphology of Nanocarriers

Hydrodynamic diameters and polydispersity index (PDI) were determined by dynamic light scattering (DLS) measurements and zeta potential by electrophoresis and laser light scattering using a Zetasizer Nano ZS (Malvern Instruments, Worcestershire, UK). Samples were prepared as described above and diluted ten times in 1 mM NaCl to achieve a constant ionic background and measured at 25 °C in clear disposable zeta cells.

The morphology of dried TMC nanoparticles was imaged with scanning electron microscopy (SEM; Jeol JSM-7001FA, Tokyo, Japan) at an accelerating voltage of 15 kV. The samples were 1000-fold diluted with water, placed on a grid, and air-dried overnight under vacuum. The grids were sputter coated with 10 nm gold under vacuum before imaging (Leica EM SCD 500, Heerbrugg, Switzerland). SWE06 and Cationorm^®^ nanoemulsions were stained with uranyl acetate and their morphology examined by transmission electron microscopy at 200 kV (TEM; Tecnai G2 T20 Sphera, FEI, Hillsboro, OR, USA).

To assess the particle area and radius, microscopic images were analyzed using ImageJ 1.46r software (National Institute of Health, Bethesda, MD, USA). Assuming that the nanostructures are roughly spherical, their radii were calculated from the particle areas, and subsequently their diameters, according to Equation (1):

(1) $$r=\sqrt{\frac{A}{\pi }}$$

#### 3.3.2. Loading Efficiency

The adsorption rate of pDNA onto nanoparticles was evaluated, measured under conditions corresponding to the *in vitro* studies as well as to the *in vivo* studies. Nanoparticle suspensions with pDNA were prepared as described above and centrifuged at 14,000 rpm for 15 min (Eppendorf 5810R, Vaudaux-Eppendorf, Basel, Switzerland). Unloaded pDNA in the supernatant was quantified by PicoGreen assay according to the manufacturer’s protocol. A calibration curve was established by plotting the fluorescence intensity of each standard against the concentration series. The samples were excited at a wavelength of 480 nm and the fluorescence emission intensity was measured at 520 nm using a fluorescence microplate reader (Tecan Group Ltd., Männedorf, Switzerland). The amount of pDNA adsorbed on TMC nanoparticles or nanoemulsions was calculated by subtracting the pDNA quantity found in the supernatants or in the oil phase after centrifugation, from the amount of pDNA initially added.

#### 3.3.3. *In Vivo* Immunogenicity of pDNA-nanoformulations

Female C57BL/6 mice (Harlan, Itingen, Switzerland) were maintained under standardized conditions in the animal facility of the University of Lausanne (Epalinges, Switzerland). The study was performed in compliance with the Swiss Federal Veterinary Office guidelines (Authorization 2475, SCAV, Lausanne, Switzerland). The formulations were prepared as described above, diluted with phosphate buffered saline (PBS) and Ag85A encoding pDNA at a final concentration of 50 μg per dose was added to the diluted nanoparticles and nanoemulsions. Eight-week-old mice (*n* = 4 per group) were immunized on Days 0, 21, and 42 with a dose of 50 μL by intramuscular administration in the hind limb. The mice were bled 1 week after the third immunization by cardiac puncture and sera were stored at −20 °C. Anti-Ag85A-specific serum IgG titers and IgG isotypes 1, 2b, and 2c were determined by ELISA at the endpoint of the optical density-log dilution curves. Non-responding mice were given an arbitrary titer of 1. Briefly, 96-well microtiter plates (Nunc, Roskilde, Denmark) were coated with 1 μg/mL rAg85A in PBS overnight at 4 °C, followed by blocking of the wells with 1% (*w*/*v*) BSA in PBS for two hours at room temperature (RT) to reduce nonspecific binding and subsequent addition of serial dilutions of serum ranging from 102 to 2.2 × 105 for one hour at RT. Ag85A specific antibodies were detected by incubating HRP conjugated goat anti-mouse IgG, IgG1, IgG2b or IgG2c (1 h, RT) and by developing plates with TMB for 5 min at RT in the dark. The reaction was stopped with 1 N sulfuric acid and absorbance was determined at 450 nm with an iMARK micro plate reader (Bio-Rad Laboratories, Hercules, CA, USA).

#### 3.3.4. Cell Culture

The murine macrophage cell line RAW264.7 was obtained from American Type Culture Collection (ATCC, Rockville, MD, USA). Cells were cultured in cell culture medium based on DMEM, supplemented with 10% heat-inactivated fetal calf serum (FCS) and 1% penicillin/streptomycin, at 37 °C in an atmosphere containing 5% CO_2_. Cells were seeded at an initial density of 3 × 10^4^ cells per well in a 96-well plate, if not indicated otherwise.

#### 3.3.5. *In Vitro* Cytotoxicity and Cytokine Release Assays

Potential cytotoxic effects of the nanoparticle formulations were evaluated by XTT assay to determine cell viability following nanoparticle exposure. RAW264.7 cells were cultured with the nanoformulations suspended in FCS containing cell culture media for 24 h. Additionally, positive controls were run in parallel, SDS 0.2% for cytotoxicity and LPS (5 μg/mL) for proinflammatory cytokine release. The cell supernatants were withdrawn for cytokine analysis, replaced by XTT reagent and incubated for another 5 h at 37 °C. The mean OD value of the wells was determined by measuring at a wavelength of 490 nm using a microplate spectrophotometer reader (Power Wave XS, Biotek, France) and corrected by the blank value. The relative cell viability was expressed as a percentage relative to untreated control cells cultured under the same conditions.

Cell supernatants from the cell viability assay as described above were centrifuged at 300× *g* for 5 min and frozen at −80 °C prior to analysis. TLR-9 and NLR-2 control samples included nanoparticle complexes, formed with pCpG-free pDNA and D-MDP. Both control ligands were applied at the same concentrations as pDNA and MDP, respectively. For NLR-2 pathway inhibition, cells were pretreated with 100 nM gefitinib for 1 h before stimulation with the nanoparticle formulations. Cell supernatants of all cytokine release experiments were diluted 1:10 and TNF-alpha induction determined by mouse TNF-α sandwich ELISA, according to the manufacturer’s instruction. Cytokine concentrations were calculated against a standard curve prepared in duplicates.

#### 3.3.6. Statistical Analysis

The statistical significance for *in vitro* experiments measuring TNF-α release, was assessed by Student’s *t*-test for two samples, assuming equal variance. Experiments were repeated at least once and data evaluated are mean values of triplicate samples. Statistical analysis was performed following logarithmic transformation of antibody titers. The statistical significance for *in vivo* data measuring IgG antibody titers was assessed by a two-tailed Mann–Whitney test (*n* = 4). The statistical analysis was carried out using GraphPad Prism 6 software (GraphPad, San Diego, CA, USA) and p values less than 0.05 were considered to be significant.

## 4. Conclusions

Polymeric TMC nanoparticles were compared with squalene-in-water and mineral oil-in-water nanoemulsions for delivery of Mtb antigen Ag85A encoding pDNA to induce antigen-specific immune responses *in vivo*. Among the three nanoformulations evaluated, TMC nanoparticles were identified as the best candidate for polarizing immune responses to Th1, which is desirable in inducing immunity against Mtb infections. Subsequently, optimization of the pDNA-nanoformulations with MDP was investigated in mechanistic *in vitro* studies. We showed that simultaneous targeting of TLR-9 by unmethylated CpG motifs present in pDNA and NLR-2 by MDP results in significantly increased proinflammatory cytokine release in a synergistic manner. In conclusion, TMC nanoparticles were shown to be promising carriers for pDNA to polarize immunity towards cell-mediated immune responses. Our *in vitro* investigations revealed the potential of co-delivery with MDP that can be taken into consideration in future *in vivo* studies to further increase immunogenicity of these DNA vaccine formulations.
